# Rheumatism

**Published:** 1858-07

**Authors:** 


### RHEUMATISM.

Prof. Lebert reported the observations he had made upon rheumatism in his medical clinic, during the years 1856–57. During the past four years he has met with two fatal cases: one of acute articular rheumatism with pleuro-pericarditis, in which death occurred with cerebral symptoms, with inflammatory congestion and purulent deposit in several of the diseased joints; the brain, however, exhibited no traces of disease. The second case was also one of acute articular rheumatism, in which the inflammation of the joints had passed into the chronic form, and in which death suddenly supervened, with indications of peritonitis, caused by a "twisting" of an intestine. The duodenum was so turned upon its axis at its union with the jejunum, as to produce complete obstruction of the bowel. The tortion was caused in part by cellular bands passing between the duodenum and pancreas, probably the result of former peritonitis; immediately under this portion, the bowel was very much contracted—reduced, in fact, to nearly one-third of its original volume. In the knee-joint were all the indications of a chronic inflammation.

Rheumatism of the spinal column usually accompanied the articular affection, and in several cases as rheumathrisis of the cervical vertebræ, a disease which is often mistaken for spondylitis, but which, however, is often readily relieved by topical bloodletting and mercurial applications. A remarkable complication was observed in the case of a boy, sixteen years of age, who had already passed through a violent attack of acute rheumatism, and was then seized with extensive periostitis in both thighs, in the course of which deep ichorous abscesses were formed, and the substance of one os femoris became so diseased, that a spontaneous fracture took place, which fully united only after the lapse of three months. The unhealthy discharge was much improved by injections of tinct. iodini. The same patient was also attacked as he was recovering from the effects of the abscess with double pleurisy, and finally with Bright's disease, and yet, in spite of all these dangerous complications, he was discharged at the close of the year 1855, much improved, the fistulous openings in the thigh still remaining.

Prof. L. employed lemon juice, and upon the whole with satisfaction, in acute rheumatism, where he had hitherto given natr. nitric (ʒii.–℥ss.), and tart. stib. (gr. i.–ii. daily in aqua ℥iv.) He has especially found it an excellent remedy in ill-defined articular rheumatism, even with large effusions into the joints. After three or four days, a gradual improvement is observed. At the end of a week, the patients are much relieved; and after a treatment of ten to seventeen days, have nearly attained a cure, which, in the course of three weeks, is complete. In some instances, a cure is much more rapid, while, in others, the remedy is inert. The heat and frequency of the pulse are very soon reduced, and the tendency to perspiration increased. The appetite returns after the abatement of the fever. Prof. L. commences with ℥iv. of the lemon juice daily, increasing the dose to ℥vi.–viii., of which a tablespoonful should be taken hourly in sweetened water. In general, this remedy is well tolerated by patients. Citric acid has also been tried by Prof. L., in doses of ʒii., iv., to ℥i. daily, but with less beneficial results; it was not so well borne as the juice. Rheumatism of a single joint was treated with greater energy in the use of antiphlogistic and derivative means; internally with iod. pot., alternate with cathartics; when the pain and swelling were in a measure reduced, the joints were painted every two or three days with tinct. iodini, frequently followed with sulphur and alkaline baths, and in obstinate cases with moxas. An energetic treatment is necessary, since a neglected inflammation of the joints often induces incurable evils, and may possibly lead to amputation.

In cases of the so-called chronic or dry arthritis (arthritis deformans), or of chronic inflammation of the periosteum and the entire epyphisis, with marked modification in the nutrition of the bones and cartilages, the internal use of tinct. iodini (gtt. xv. ter die as largest dose) proved the best anodyne.

